# Financial access and women's role in household decisions: Empirical evidence from India's National Rural Livelihoods project^☆^

**DOI:** 10.1016/j.jdeveco.2022.102821

**Published:** 2022-03

**Authors:** Anjini Kochar, Closepet Nagabhushana, Ritwik Sarkar, Rohan Shah, Geeta Singh

**Affiliations:** a Stanford University and International Initiative for Impact Evaluation, USA; b Vrutti Livelihood Impact Partners, India; c International Initiative for Impact Evaluation, India

**Keywords:** Women's decision-making, Self help groups, Loan size

## Abstract

Government programs supporting self help groups (SHGs) generally target women on the assumption that doing so enhances women's decision-making. The empirical evidence, however, is mixed. We advance and test one explanation: the loan amounts offered by most SHGs may be too small to impact women. Our analysis is based on SHGs developed under India's National Rural Livelihoods Mission, a program that supported both small loans from internal savings and larger loans through Community Investment Funds (CIFs). Exploiting variation in their phasing and amount, we document a large effect of CIFs on women's decision-making and on intra-household allocations.

## Highlights

• The impact on women's decision-making of their improved access to loans depends on loan size.
• Targeting women for financial access provides no additional benefit if loan sizes are small.
• Our analysis uses variation in implementation of a national program for Self Help Groups in India.
• The results suggest the importance of implementation for the success of the program.

## 1 Introduction

The mixed success of micro-finance programs in reducing poverty mirrors similar variation in their impact on women's role in household decisions, despite the fact that most group-based approaches to financial inclusion target women. A review of several microfinance programs finds insignificant effects on women's decision-making ability in three out of the four studies that evaluated this outcome (Banerjee et al., 2015c). Similarly, recent systemic reviews of programs that support women's Self Help Groups (SHGs) suggest an average effect on women's decision-making that is small and frequently insignificant (Brody et al., 2017; Jayachandran et al., 2020).^1^ For example, Jayachandran et al.‘s review of livelihoods-focused SHGs reveals positive effects on women's decision-making ability in only half the studies they examined.

One explanation for the marginal effects of micro-finance programs comes from theoretical models of poverty traps.^2^ These models postulate that movements out of poverty require a “big push” that generates a large jump or change in the resource base of poor households. Responding to this, governments are increasingly embracing programs that grant ultra-poor women livelihood-generating assets, while also providing them with training to upgrade their skills and other complementary inputs. Can big push programs enable changes in women's status? And, if so, are there less costly ways of making this push that may enable much wider coverage of all below-poverty-line women, not just the ultra poor?^3^

We address this question by examining the impact of large increases in loan amounts to women who are members of Self Help Groups formed and supported under India's National Rural Livelihoods Project (NRLP), a program intended to enhance women's livelihoods through a federation of community institutions with SHGs at the lowest level. To evaluate NRLP we, along with other collaborators, implemented a cross-sectional survey of approximately 15,000 SHG members across 8 of India's poorest states in 2019, as the program approached its end.^4^ The data thus enable an impact of the program at scale, after it had reached almost universal coverage of its targeted 70 million below-poverty-line households spread across 600 districts and 600,000 villages. The survey was designed to identify the impact of NRLP exploiting its phasing across blocks and, within blocks, across villages of the country, and provides a wealth of information on households and SHGs. The considerable variation in SHG age and characteristics in the sample generates corresponding variation in the program inputs provided to SHGs, variation that is rarely available in studies based on pilots of programs in a particular state.

As in other such programs, membership in a NRLP SHG requires regular savings. Loans, in early stages of a SHG's life cycle, are based on the group's accumulated internal savings. Given small monthly savings amounts, SHGs' savings accumulate very slowly. Amongst survey SHGs, monthly savings varied from INR 10 to INR 100, with a mean of INR 30 and a median of only INR 10. Correspondingly, initial loan amounts are small: The average loan size across all SHG members in the first year of formation for SHGs in our sample (formed between 2012 and 2018) is just INR 1,014, with this number increasing to INR. 4150 if the sample is confined to those who borrow. This latter amount is equivalent to wage earnings from approximately 20 days of work.^5^ At this level, it is unlikely that the loans offered by the program would significantly impact women's economic position within the household.

NRLP, however, promoted an institutional architecture that magnified the economic benefits to members over time. This was primarily through a grant-in-perpetuity to SHGs, the Community Investment Fund (CIF), a grant that doubled average loan amounts to individual members. Repaid funds remained within the SHG federation to support continued internal lending, and hence provided the basis for a significant improvement in incomes. Though the central government set guidelines for the CIF amount per SHG, each state had the discretion to independently determine funding amounts. The variation in state funding per SHG is significant, ranging from INR 30,000 to INR 110,000.

We evaluate the impact of CIFs on an index of women's decision making using a difference-in-difference methodology that exploits variation in the incidence of treatment at the SHG level and state-level differences in the amount of the grant. Our approach follows Duflo (2001), Jacoby (2002) and others who similarly use cross-sectional variation in program intensity to identify program effects through difference-in-difference regressions.^6^ We compare outcomes for SHGs that did and did not receive CIFs in high-CIF states relative to this same difference in low-CIF states. This controls for possible endogenous variation in program intensity and incidence caused by factors such as the targeting of “superior” SHGs or the provision of larger CIF amounts in states with better implementation. Our ability to identify the impact of CIFs is enabled by the large number of states covered in our survey and by the extensive phasing of the program across blocks and villages. The former ensures significant variation in CIF amounts per SHG while the latter generates variation in the incidence of CIFs across SHGs, with variation across both dimensions being critical for identification.

In addition to tests of the identification assumptions underlying a difference-in-difference regression, we also subject our results to a broad set of falsification tests, including tests of whether the results merely reflect differences in socio-economic conditions across states. We find strong evidence that these large improvements in a woman's access to financial resources enhance her decision-making role within the household.

We extend our analysis to address the policy relevant question of whether targeting women for financial inclusion generates benefits beyond those that would obtained by improving financial access for households, without regard to who within the household has access to loans. Building on a large body of research that tests the unitary or income-pooling model, we confirm that women's financial access to CIF funds affects household consumption allocations, even in regressions that condition on total household savings. This evidence supports the view that women's access to large loans constitute a “distribution factor,” shifting household preferences. We find, however, that this result applies only to large loans: women's access to the significantly smaller loans available after one year of internal SHG savings, without access to CIFs, affect consumption allocations only through their effect on household savings. That is, providing women with access to small loans yields results that are similar to improvements in the overall financial position of the household, implying no benefit from targeting women.

Our research builds on theoretical models of inter-temporal household decision making that illustrate how women's bargaining weights are updated over time as a consequence of changes in their reservation utility (Mazzocco 2007; Voena 2015). Within this context, Chiappori and Mazzocco (2017) forcibly make the point that improvements in women's bargaining weights will only result from interventions large enough to affect women's (autarkic) well-being in the event that the marriage breaks down. The small loan amounts provide by SHGs and microfinance groups in early years are unlikely to meet this threshold, providing one explanation for why several studies that evaluate SHGs even after a period of 3 years or so find their impact to be limited. Evidence of larger impacts primarily comes from programs that combine the normal savings-and-lending function of SHGs with additional interventions such as changing social norms and hence are more likely to meet the theoretical criterion required for the program to affect women's decision-making (Jejeebhoy et al., 2017; Brody et al., 2017; Bandiera et al., 2020).

As previously noted, our research is also related to the growing evidence on multifaceted graduation programs that provide large grants, training and other complementary inputs to ultra-poor households (Banerjee et al., 2015a). In general, these studies report large and sustained improvements in asset ownership, income and expenditure as do evaluations of other programs that provide large cash transfers to households. For example, Egger et al. (2019) and Haushofer and Shapiro (2016) establish the effect of large cash transfers by the NGO GiveDirectly on Kenyan households, while Angelucci, de Giorgi and Rasul (2018) provide evidence that transfers in Mexico's Progresa program enables improvements in investments in networks characterized by strong ties. Whether these large transfers improve women's decision-making is an open question. The review of six Ultra Poor Graduation programs in Banerjee et al. (2015a) suggests no impact three years after the program was introduced, though there appeared to be some early gains in a first endline study conducted two years after the baseline. Roy et al.’s (2015) evaluation of Bangladesh's program reports mixed results, with greater control by women over the assets created directly by the program (livestock), but not other assets, and reduced involvement by women in decisions relating to income, purchases for themselves and household budgeting.

Even if the effect of large cash transfer programs on women's empowerment were positive, their cost suggests a limited potential to impact outcomes on scale; though BRAC's program covered 360,000 households by 2014 and was intended to reach 650,000 households by 2016 (Bandiera et al., 2017), these numbers are small relative to that of NRLP. By 2020, NRLP reached over 72 million households through 7 million SHGs, suggesting the ability to transform women's status if successful features of the program can be ensured and sustained in all SHGs.

This, of course, remains the challenge. Our analysis identifies the impact of CIFs by exploiting variation in their amount and timing across SHGs, that is, variation in how the program was implemented across India's states. Thus, the challenge to policy is ensuring that features of the program's design that enable a sizeable impact on women are implemented in full and not scaled back to a level where their effect is negligible.

The rest of this paper is organized as follows. Section 2 describes the conceptual framework underlying our empirical analysis and the use of a decision making index to evaluate the program's impact on women's bargaining power. The next section discusses India's National Rural Livelihoods Project and its implementation. Details of the survey data, data definitions and summary statistics are in Section 4 while Section 5 describes the empirical framework. Section 6 contains the empirical results of this paper. Section 7 concludes.

## 2 Women's status and intra-household decision-making

The empirical analysis of this paper evaluates the impact of large SHG loans on one aspect of women's empowerment, an index of their input in household decisions regarding consumption expenditures. In this section, we discuss the theoretical underpinnings for the focus on women's decision-making role, noting its limitations and advantages. This theoretical literature carries implications for the policies that can affect this role. We close with a brief discussion of these implications.

The need to improve the economic, political and social standing of women is well recognized particularly in economies such as those of South Asia where a patriarchal culture has long discriminated against women. These economies have historically been characterized by significant differences in women's access and ownership of assets, including education and health (Chen et al., 1981). It is widely agreed, however, that empowering women requires attention not just to their ownership of assets but also to their ability to exercise choice, to set their own goals and to act on them (Kabeer 1999). This may require interventions that inform and educate women about their rights, entitlements and choices, prior to providing them the means to exercise these rights. And, it assumes that, given such knowledge, there exists at least a subset of goods for which women's preferences differ from those of men.

The concern with this aspect of women's empowerment, the extent to which women can make choices over the goods and services that matter to them, dovetails with theoretical models of household behavior that extend the traditional unitary or common preference model to allow for differences in preferences amongst members and for individual choice within the family unit. Within the context of a common preference model, questions regarding who makes decisions within the household do not arise. In contrast, models that allow for differences in preferences between husband and wife require a stand on how consumption allocations are decided. The “collective” model (Chiappori, 1988; Browning and Chiappori 2006), for example, assumes that households make decisions efficiently. Under this assumption, the household's utility function represents a Pareto-weighted sum of the utility functions of its individual members, with a woman's relative Pareto weight reflecting the extent to which her preferences affect household allocations.

This model is most commonly tested by examining whether a woman's individual income affects expenditures in regressions that control for total household income (Thomas 1990; Bourguignon et al. 1993; Attanasio and Lechene 2014). Under a set of identifying assumptions, this constitutes a joint test of common preferences and the role of women's income as a distribution factor and hence as a determinant of her relative bargaining weight. These assumptions are sometimes difficult to support. For example, in the context of this paper, if a woman's access to loans affects her reservation wage rate, one would have to restrict attention to goods that are separable from leisure. Most goods, including food items, would fail to meet this criterion, given the significant amount of women's labor that goes into home production.

Alternatively, one could directly test the impact of any given policy on women's bargaining power, should a measure of her Pareto weight be available. As Chiappori and Mazzocco (2017) note, a woman's relative Pareto weight can be interpreted as a measure of her relative decision power, taking the value zero if a woman has “no say in household decisions” and the value one if she has “perfect control over the choices made by the household (p. 999).” Building on this, a growing empirical literature tests the impact of policies on women's bargaining power using indices of decision-making ability. These indices are typically derived from survey questions that query women about their role in decisions regarding expenditures on health, education, food and other consumption items (Anderson and Eswaran 2009; Tarozzi et al., 2015; Banerjee et al., 2015b; Angelucci et al., 2015; Karlan et al., 2017; Hoffmann et al., 2021).

The validity of this approach depends not just on how well a given index measures a woman's bargaining power but also on the relationship between decision-making and the distribution of preferences.^7^ With reference to measurement, research suggests that men and women may differ in the extent to which they relate decision making to autonomy, and that these interpretational differences vary across countries and cultures (Seymour and Peterman 2018). Results may also be sensitive to the questions asked and the set of goods over which the index is defined (Kabeer 2001). The second concern reflects the possibility that time spent on making decisions may represent a component of preferences, responding to bargaining weights rather than simply serve as a measure of these weights. For example, if the time spent on decision-making reduces utility, then husbands with greater bargaining power may allocate all decisions to women, knowing that women will make choices that reflect their husband's preferences more than their own. If this is so, then improvements in a woman's bargaining power may *reduce* her involvement in household decisions if she chooses to delegate more of them to her husband.

Thus, studies that assess the impact of improvements in women's resources on indices of her role in household decisions may yield results that differ from tests of the income pooling hypothesis, rendering difficult their interpretation. While qualitative evidence on the import of decision-making can help in interpretation (Daminger 2019), such evidence is rarely available in large quantitative studies. Lacking this, we support our primary results based on a decision-making index with tests of whether women's access to CIFs constitute a distribution factor, affecting consumption allocations in regressions that condition on (total) household resources. The combination of results from these two approaches, if complementary, provide strong evidence of the impact of CIFs on women's decision-making and help to interpret this finding.

Our research contributes to both these strands of the empirical literature in our concern for the importance of loan *magnitudes* in the determination of bargaining weights. The literature on intertemporal intra-household decision making under limited commitment that allows for the evolution of bargaining weights over time suggests their importance (Mazzocco 2007; Chiappori and Mazzocco 2017; Voena 2015; Kocherlakota 1996; Ligon et al., 2002; Ligon 2002). At the start of the marriage, bargaining weights reflect a set of distribution factors that in turn help determine each partner's reservation utility or the utility obtained in the event of the breakdown of the marriage. In a dynamic framework with limited commitment in which partners are unable to commit to future plans, efficiency requires satisfaction of a set of participation constraints that ensure that each partner is at least as well off within the marriage as they would be should it break down. Correspondingly, improvements in the reservation utility of any partner require an updating and revision of bargaining weights, with corresponding changes in household outcomes.

This framework carries several important implications for the design of policies intended to empower women. First, it suggests that programs and transfers will improve a woman's bargaining position only if they continue to apply or if the woman remains eligible should the marriage break down. Membership in a SHG and the loans that members can access belong in this set, since membership is not tied to marriage. In contrast, policies that provide women transfers or income at stages of their lifecycle *within* a marriage, such as grants to women in India for the institutional delivery of a child or grants in cash or kind for pregnant women will not have such an effect. Second, a woman does not need to borrow to realize the effect of her improved access to financial resources. It is the possibility of access to large loans that enhances her position within the household. However, for women who *do* borrow from the SHG, membership affects household savings as well as the woman's bargaining position.

A third implication of this framework, one that underlies the analysis of this paper, is that updates in (initial) bargaining weights will occur only if changes in individual reservation utilities are large enough to cause participation constraints to bind, that is, to make an individual's threat point viable (Chiappori and Mazzocco 2017).^8^ Small improvements in access to credit are unlikely to have this effect. That is, while the small loan amounts provided against the SHG's internal resources will enhance the savings of member households, these loan amounts are unlikely to affect a woman's intra-household bargaining power.

## 3 The program and its implementation

The analysis of this paper is based on India's National Rural Livelihoods Project (NRLP), a program intended to enhance livelihoods through the formation of Self Help Groups (SHGs) comprising women from amongst the country's poorest households.^9^ While the primary goal of the program was to “reduce poverty through promotion of diversified and gainful self-employment and skilled wage employment opportunities” (Government of India 2017, p.1), it also emphasized the importance of enhancing women's status. Thus, the same document (p.7) notes that “well-nourished SHGs and their federations would emerge as institutions facilitating diversification of livelihoods and empowerment of women.”

NRLP was implemented in a phased manner. The program was initiated in 2012 in a set of identified “early blocks,” the four poorest blocks in the four poorest districts of each state. Implementation in “late blocks” of the same districts generally followed with a lag of approximately 4 years. Within each block, the process of SHG formation was undertaken at the level of a “cluster,” a program-specific administrative unit comprising approximately 25 villages. Cluster-level project teams moved from one village to another following a stipulated village entry plan until a first round of SHG formation had been completed. This process was repeated in successive rounds until the entire target population had been covered.

Project teams responsible for forming a SHG did so by identifying hamlets or narrow residential groupings of relatively disadvantaged households and then intensively working within the hamlet to form groups of approximately 10 women. After group formation, the project team trained members on SHG principles, including a set of five norms or rules, referred to as *Panchsutras* (regular meetings, regular savings, regular lending, regular repayment and the maintenance of books of account) which formed the basis of the program's evaluation and assessment of the quality of SHGs.

In early stages, the program's focus was on accumulating savings of members and “internal” lending based on these savings. Groups could select the amount that each member was required to save each month. These amounts were low: The mean amount across the approximately 4700 SHGs we surveyed was INR 30 per month (40 US cents), with the median amount being just INR 10. Not surprisingly, initial loans were small. SHGs formed in 2018 reported an average loan amount of just INR 2415 (approximately US $33, in 2020), with this amount being INR 7015 for older SHGs formed in 2015 and INR 6998 for those formed in 2012.^10^

However, NRLP recognized that moving households out of poverty required additional resources. These came from two sources. The first was a “Revolving Fund” (RF), a relatively small grant of INR 15,000 that each SHG was eligible for once it had demonstrated at least 3 months of success in maintaining the 5 *Panchsutra* principles. The second was a much larger grant in the form of “Community Investment Funds” (CIFs). This was provided “in perpetuity” to SHGs approximately 6 months to a year after their formation.^11^ Program guidelines suggested CIFs of INR 110,000 per SHG. However, states had the flexibility to determine this amount resulting in the significant state-level variation in CIFs discussed in the Introduction. Amongst other factors, this variation may reflect differences in state priorities regarding the program and hence corresponding differences in the level of resources committed by the state: Though the Central government provided the majority of program funds, state governments contributed a significant share.^12^

While the attempt to ensure access to large loans recognizes their importance for sustained improvements in poverty, an acknowledgement of the importance of ownership came mainly in the form of the requirement that SHG membership be restricted to women. Correspondingly, loans were provided to female members of the household as were training sessions on financial literacy and on vocational and life skills. However, while training is likely to “stick” to the individual who receives it, the same does not necessarily apply to control over SHG loans. Guidelines for NRLM suggested that state governments assist SHG members lacking bank accounts to avail of the benefits of a program launched in 2014, the Jan Dhan Yojana, that aimed at providing each household access to a basic savings account (Government of India 2017). However, Jan Dhan Yojana targeted households, rather than women within the household. And, correspondingly, though the program required each *SHG* to have a bank account, there was no requirement that individual members should have their own accounts. That is, loans from SHGs to members did not have to be deposited in individual bank accounts, the method generally used to ensure women's control over transfers targeted at them. Thus, the question of whether women could control the loans they received is particularly germane in this context.

NRLP's emphasis on providing training to members on financial and other outcomes and on ensuring the quality of SHGs through continuous oversight required commensurate growth in the program's administrative capacity. A hallmark of the program is a set of unique institutional arrangements put in place for the purpose. First, NRLP supported a federation of institutions, with SHGs at the lowest level. SHGs within a village were, over time, federated into village level organizations (VOs), and then into Cluster Level Federations (CLFs). This federated structure was intended to overcome the problems of delivering inputs, including training and market access, to large numbers of small SHGs, while simultaneously providing a means of monitoring and ensuring the quality of each SHG. Equally important, NRLP supported an innovative program that recruited women, at the time of program entry into each village, to a “community cadre.” Following an extensive two year period of training, this cadre enabled an expansion in the number of teams entrusted with the formation of SHGs, VOs and CLFs and hence the growth of the program at the extensive margin. It also enabled the program's growth at the intensive margin or in the quality of each SHG, with cadre members monitoring the operations of SHGs, certifying their quality and providing them the training to effectively access and use program resources including CIFs. Members of the community cadre were responsible for training SHG members in the preparation of micro-investment plans and for certifying the SHG's adherence to the *Panchsutras*, requirements for the receipt of CIFs (Government of India 2017).

Had these guidelines been adhered to, only high quality SHGs should have been provided CIFs. However, our data reveal that adherence to these principles was minimal. While 37% of SHGs reported receiving CIF funds, only 12% report having prepared a micro-investment plan. Similarly, adherence to the *Panchsutras* was low: the aggregate score of surveyed SHGs in terms of their adherence was just 2.5 out of 5. Annual Action Plans prepared by each state and our discussions with government officials suggest that these implementation failures as well as delayed receipt of CIFs were primarily a consequence of weak administrative capacity in the form of insufficient numbers of community cadre members. Thus, program implementation was characterized by a vicious cycle: Slower-than-anticipated progress in the formation of SHGs in early years delayed the recruitment of the community cadre, further slowing the growth of the program at both the extensive and intensive margins.^13^

## 4 Survey data, data definitions and sample statistics

### 4.1 Survey data

Our survey data comes from a large cross-sectional survey of SHGs and their member households across 8 states designed for an evaluation of NRLP.^14^ The study was commissioned at the end of the project, and hence lacked baseline data on households or SHGs. Instead, exploiting details of the implementation of the program, it identified program effects based on its phased introduction across blocks and, within each block, across villages.^15^

The survey sample was drawn from districts with at least two early and two late blocks (identified by each state's implementing institution, the State Rural Livelihood Mission or SRLM). Within each of these blocks, two clusters were selected using MIS data that identified project clusters and provided information on the year of village entry and SHG formation for the census of all SHGs in the district. In each cluster, two “early” and two “late” villages were chosen, with early SHGs defined as those entered in the first year and late villages being those entered in the last year of SHG formation in the cluster. Within each village, two SHGs were randomly selected from the set of those formed at the time of initial village entry for administration of the household survey to its members. A SHG module was also completed for four additional SHGs in the village, randomly drawn from SHGs listed in the MIS. The survey sample, therefore, does not constitute a random sample of SHGs, comprising instead of SHGs, in each cluster (and hence block) formed in early and late stages of the program.

The fact that the study was implemented towards the end of the program allowed the household instrument to be canvassed on SHG members, eliminating the low take-up rate that normally affect evaluations that identify households prior to the implementation of the program and hence prior to SHG formation (Banerjee et al., 2015). Equally importantly, it enabled the collection of data on the SHGs that households were members of, and hence the linking of household outcomes to SHG inputs and characteristics. This allows us to evaluate the impact of specific aspects of the program, such as the provision of CIFs, at the level of the household, exploiting rich data on each SHG such as the year of its formation and the year of receipt of CIFs.

The sample for this paper is restricted to households who were members of functioning SHGs and those formed after 2012, covering SHGs formed between 2012 and 2019.^16^ Because of the focus of this paper on women's decision making we exclude households without any adult male member, since women in these households, not unexpectedly, are generally the primary decision makers.^17^ The sample size for the major regressions of this paper that include a basic set of control variables is 12,527 (12,548 without controls). This falls to 11,967 in regressions that use an expanded set of household control variables. The sample size for regressions based on SHGs is 2568.

### 4.2 Survey modules and data definitions

Our survey instruments included a household survey, a woman's survey administered to one prime-age woman in the household, a village module and surveys of SHGs, VOs and CLFs. The household and woman's survey were intended to be administered to all members of the two SHGs in each village selected for inclusion in these surveys. However, budget and time constraints limited interviews to households who were available at the time of the survey, yielding an average of 5 members per SHG, with three or more members being surveyed in approximately 80% of SHGs.

The basic design of the household module followed that of other surveys, such as India's National Sample Surveys, providing information on household rosters and including a standard expenditure module. A distinguishing feature of our survey, however, is its collection of detailed income data on all family enterprises, agricultural and non-agricultural. This includes data on marketed and non-marketed outputs, family and hired labor used in production, and all purchased and home-provided production inputs. This depth of information enables estimates of income and hence of savings as the difference between income and expenditures, enabling regressions that correctly incorporate life cycle dynamics by conditioning on household savings.^18^ The household module also provides detailed information on all loans that were outstanding at the time of the survey, as well as on all loans that were fully repaid in the three years prior to our survey. Loan details included information on the year in which it was taken as well as its source, allowing us to construct a pseudo panel of loans from SHGs for each household extending back to 2012 and even earlier. Matching villages, village governments (Gram Panchayats) and blocks to the 2011 Indian Census provides data on population totals for each of these geographic levels, as well as data on women's labor force participation prior to the initiation of NRLP in 2011.

Our empirical analysis is enabled by additionally matching the survey data to the Project's MIS and to other data on the program. The division of states into groups based on state norms regarding CIF amounts uses information from each state's Annual Action Plans from 2013 to 2018.^19^ These Plans stipulate state-specific targets for the number of SHGs to be provided with CIFs and the total amount of CIF to be disbursed in each year and hence the amount of CIF to be provided to each SHG. These data demonstrate little variation, for any given state, within a year. Correspondingly, using state averages, we divide states into *high* and *low* intensity CIF states, based on the median CIF amount.

As noted in the preceding section, the receipt of CIFs was significantly affected by the size of the available community cadre and hence the scale of the program at the time of SHG formation. This suggests the need to control for program scale in order to identify the effect of CIFs. MIS data allows us to construct measures of scale specific to each SHG. Given that community cadre members were required to have completed a minimum of one year of active membership in a SHG, and that their training required an additional 6 months to a year, we develop measures that reflect the scale of the program two years prior to the formation of each SHG. These measures are: the number of SHGs in blocks of the district other than that in which the SHG was located, the number of villages entered in these blocks, and the number of villages entered in other clusters of the same block. These measures thus exclude data from the cluster and the block in which the SHG was located and, as noted above, relate to the scale of the program two years prior to the formation of the SHG in question.

### 4.3 Measuring women's decision making ability

We follow the literature in measuring women's decision making based on their answers to a series of questions related to their role in household decisions (Angelucci et al., 2015; Karlan et al., 2017; Banerjee et al. 2015).^20^ The survey asked a randomly selected woman in the 20–50 age group (or an older woman in households without women in their prime) about her role in decision making on a set of 26 outcomes.^21^ Specifically, the question asked was: “What do you think is your input into [DECISION]?” The five options (1 = little or no input; 2 = some input; 3 = equal input; 4 = mostly my input; 5 = entirely my input) are ranked in terms of an increasing role for women, so that a higher mean score reflects greater involvement in household decision-making. We use this mean value as our primary measure of women's decision-making in our empirical analysis.

Women were given the option of not responding to a question if the item was not relevant to them. Averaged across all decisions, the non-response rate was just 5.95%. However, the non-response rate was high (18%) for two items, decisions regarding the marriage of a child and the sale of agricultural land. It also averaged 13% for loans from moneylenders and from banks. The non-response rate for all other items was less than 10%, averaging 4%. To ensure that our results are not driven by variation in the non-response rate, we also report regressions that calculate the decision-making index excluding decisions regarding these four items.

As noted in Section 2 of this paper, the use of any index is subject to questions regarding interpretation and measurement error. While the quantitative nature of our survey^22^ and the limitations of the data precludes us from addressing these issues, we attest to the robustness of our results by reporting results using different methods to compile the index. In addition to indices that omit items with a high non-response rate, these include an index derived using principal components as well as an index constructed using the top two responses (mostly/entirely my input). For the latter, a score of 1 was assigned (to each outcome) if the woman stated that she was fully or primarily responsible for decisions. Scores on individual items were then aggregated into a total percentage score*.* Finally, reverting to the mean score, we also report results for some individual questions (expenditures on food and decisions regarding what food to cook), and sub-groups of the commodities considered in the overall index. These sub-groups are: decisions regarding investments in children (school choice, expenditure on their health); clothing (for the woman, her husband and children); household durables (TV, cell phones etc, but also expenditure on home improvements and the construction of toilets); and loans (from all sources, as well as decisions regarding loans to her family and her husband's family).

### 4.4 Summary statistics

**Fig. 1:**
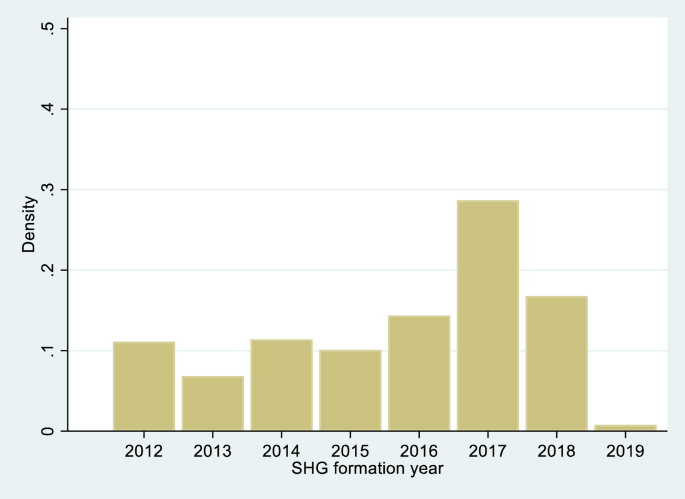
SHGs by year of formation.

**Fig. 2:**
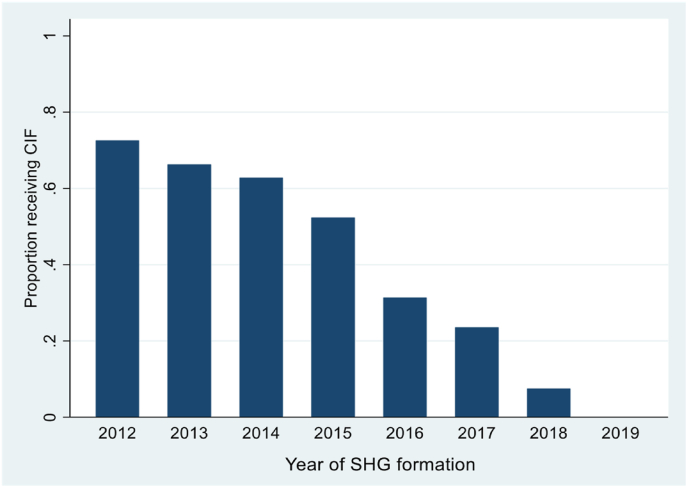
Proportion of SHGs receiving CIF by year of SHG formation Sample: full sample of SHGs, n = 4804.

**Fig. 3:**
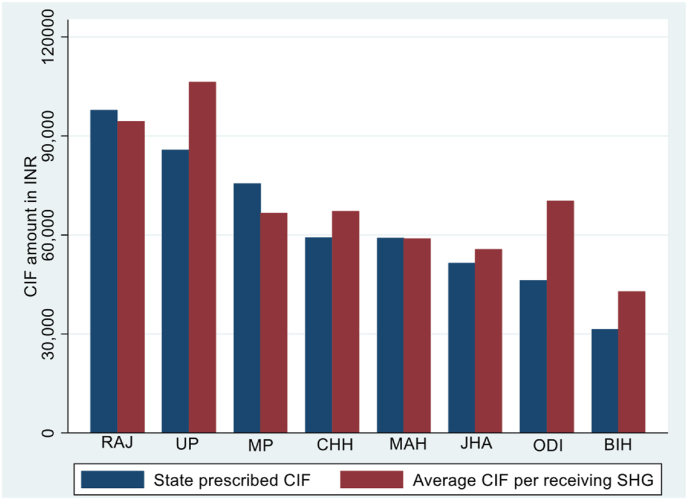
Average amount of CIF by prescribed by States and average amounts reported per receiving SHG.

We commence this section with a set of figures that demonstrate the variation that we exploit in this paper. Fig. 1, a histogram of SHGs by their year of formation, reveals the range in this variable amongst survey SHGs, with the year of formation varying from 2012 to 2019. Fig. 2, Fig. 3 describe the variables that serve as the basis of our identification strategy, specifically the proportion of SHGs reporting receipt of CIFs and variation in the state prescribed CIF amounts per SHG. Not surprisingly, the proportion of SHGs reporting receipt of CIF increases with the age of the SHG, being largest for SHGs formed in 2012 and falling for newer SHGs (Fig. 2). However, this figure also reveals the significant variation in CIF receipt across SHGs of the same age. Thus, amongst SHGs formed in 2012, as many as 23% report that they had yet to receive CIFs. This number increases to 44% of SHGs formed in 2015.

Fig. 3 documents the extensive variation in the CIF amounts per SHG prescribed by each state, and the close correlation between received amounts and prescribed amounts. States that prescribed relatively large amounts are headed by Rajasthan, closely followed by Uttar Pradesh, and include the relatively poor state of Madhya Pradesh. In Rajasthan, the state dictated that each SHG should receive an average of INR 100,000 in CIF funds.^23^ In contrast, Bihar recommended a CIF of only INR 30,000 per SHG. The 4 states with the least CIF funds include Maharashtra, a relatively wealthy state. These 4 states (Bihar, Jharkhand, Odisha and Maharashtra) comprise the “low intensity” states, states with the average CIF amount per SHG being below the median for all states. Correspondingly, the states of Rajasthan, Uttar Pradesh, Chattisgarh and Madhya Pradesh constitute the “high intensity” group. The variation in CIF funding across states does not closely correlate with measures of state development, a point that we substantiate in our regression analysis.

**Fig. 4:**
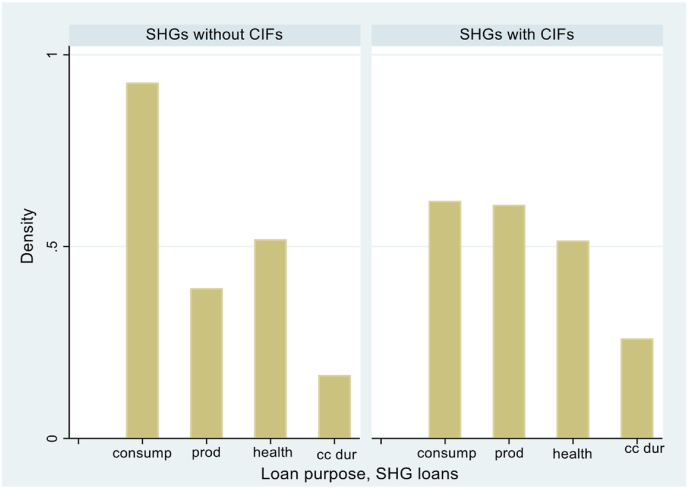
Purpose of SHG loans, SHGs with and without CIFs.

While we provide descriptive evidence of the impact of CIFs on the size of SHG loans later in this paper, Fig. 4 graphs their impact on the use of loans. In SHGs that have not yet received CIFs, the vast majority of loans to members (60%) are used to finance daily consumption needs with only 16% of loans being used for productive investments in agriculture, livestock and other family enterprises. Following receipt of CIFs, this latter number jumps to 25%, with loans for daily consumption needs falling to 46%. There is little change in the proportion of loans used either for health purposes or for the purchase of consumer durables. This change towards productive loans suggests an impact of CIFs on household incomes.

Table 1 provides summary statistics of the main variables used in our regression analysis, including the decision-making index (*dmindx),* characteristics of SHGs, and of the villages in which households are located. The data are provided for the full sample, and then separately by high and low intensity states, and by SHGs with and without CIFs within each of these state groupings.

**Table 1:** Summary statistics.

Variable	Full sample	High intensity states		Low intensity states	
		With CIF	Without CIF	With CIF	Without CIF
*Household variables*					
Decision making index (percentage)	3.08 (0.61)	3.03 (0.60)	2.98 (0.61)	3.20 (0.61)	3.14 (0.59)
Prop. SC/ST	0.32 (0.47)	0.30 (0.46)	0.28 (0.45)	0.49 (0.50)	0.30 (0.46)
highest years education women	4.83 (4.93)	5.38 (5.13)	5.06 (4.98)	4.06 (4.73)	4.67 (4.82)
Household size	5.31 (2.04)	5.40 (2.01)	5.20 (1.93)	5.58 (2.07)	5.23 (2.12)
Mean agricultural land holding (acres)	1.23 (2.50)	1.04 (1.67)	1.21 (2.03)	0.88 (1.67)	1.49 (3.31)
Household savings (INR ‘000)	−44.76 (103.37)	−45.17 (101.51)	−48.25 (103.71)	−39.93 (102.47)	−43.94 (104.42)
Clothing share of annual expenditures (%)	6.63 (4.38)	6.64 (4.51)	6.43 (4.34)	6.40 (3.97)	6.87 (4.48)
*SHG and geographic variables* GP					
Village population	2629.19 (2774.04)	2138.00 (2031.41)	1433.20 (1172.41)	4023.71 (3465.15)	2735.84 (2879.50)
Distance to block capital	16.41 (15.30)	19.63 (14.66)	20.29 (17.52)	12.54 (12.61)	14.76 (14.47)
SHG size	11.44 (1.46)	11.48 (1.45)	11.10 (1.39)	11.66 (1.40)	11.54 (1.51)
SHG prop. SC/ST	0.63 (0.43)	0.58 (0.44)	0.61 (0.44)	0.66 (0.41)	0.65 (0.42)
SHG monthly savings rate	26.64 (89.53)	22.26 (27.64)	25.08 (32.49)	24.60 (145.49)	31.74 (80.29)
Sample size – households	12,527	2490	3464	1922	4651
Sample size - SHGs	2568	376	707	677	808

The mean value of the decision-making is 3.08 (out of a maximum score of 5). It is higher in low intensity states and in SHGs with CIF than in those without. These differences are the basis of our identification of the effect of CIFs on women's decision-making and we discuss their implication in detail below.

The data also reveal the relative poverty of sample households. An average of 32% of households are from scheduled castes and tribes. The highest level of schooling reported by a prime-age woman in the household, at 4.8 years, is marginally short of the 5 years required to complete primary school. Approximately 66% of households in our survey own some agricultural land, with mean agricultural holdings being just 1.23 acres.

For the average household, savings are negative, indicating a dependence on borrowing to finance the deficit in incomes relative to expenditures. Expenditures on items such as clothing are a relatively small proportion of total household expenditures (7%), primarily because of the dominance of food expenditures. The data on SHGs reported at the bottom of the table comes from the set of SHGs covered in our survey (approximately 6 SHGs per village). These data reveal that the average SHG comprises 11 members, with 63% of members being from scheduled castes and tribes. The mean monthly savings rate is INR 27.

### 4.5 The difference in decision making across states and SHGs

Table 1 also reveals the pitfalls in evaluating the impact of CIFs using either a simple difference estimator, that compares treatment and control SHGs or SHGs across high and low program intensity states, or a simple difference-in-difference estimator that utilizes both these differences but without additional controls. The table reveals that women's involvement in household decisions is lower in high intensity states and that the difference in the decision making index across treatment and control SHGs in these states is *smaller* than this same difference in low intensity states. If the program is assessed just on the basis of these differences, it would lead to the conclusion that women's decision making falls with access to large CIF funds. However, such a comparison does not control for differences within states in the low and high intensity groups, an important difference given the variation in the implementation of the program across states. It additionally bundles the effect of CIFs with differences in the duration of exposure to SHGs, the primary determinant of the phasing of CIFs, and with other aspects of program implementation including variation in its scale across blocks and districts. Our regression approach, described below, controls for these differences.

## 5 Empirical methodology

### 5.1 Establishing the impact of CIFs on SHG loans

The hypothesis we test in this paper is premised on the assumption that CIFs provide a large infusion of funds into SHGs, allowing larger loans than were possible earlier and providing the basis for significant improvements in women's decision-making. We therefore start our empirical analysis by providing evidence of the impact of CIFs on the amount of SHG loans using survey data on all current loans and on those that were fully repaid in the three years preceding the survey. Using data on the year in which each of these loans was originated, we construct a panel data set of loans (by source) for each household commencing from 2012. This enables a graphical representation of mean loan amounts in the years preceding and following the receipt of CIFs. To implement this regression, we normalize each loan year by the year of CIF receipt (*year – cif_year).* We then run a standard “normalize and pool” regression of loan amounts on indicator variables for the number of years from receipt of CIFs, with the coefficients on each year representing the mean borrowing in that year.^24^

Our intent is to graphically describe mean loan amounts in periods before and after the receipt of CIFs; due to data limitations, these regressions do not estimate the causal impact of CIFs on loan amounts. The data are obtained by recall and restricted to loans received or closed in the three years before the survey. Additionally, causal identification requires dealing with the sample selection issues that arise because the set of SHGs used in the estimate of each parameter value varies: The full sample of SHGs informs estimates only for loans received in the [−1, 1] interval, that is, in the same year as the CIF or one year prior or following receipt.^25^ However, despite these limitations, this exercise is still useful in establishing the variation in loan amounts in years around the receipt of the CIF, and is particularly informative of variations in loan amounts immediately around the time of receipt of CIFs.

The regression we estimate for the amount borrowed by household i associated with SHG g in state s and year t is:

(1) $$Loan\;amt_{igst}=\delta_{0}+\overset{4}{\underset{j=-4}{\sum }}\delta_{j}1\left(\tau_{gst}=j\right)*high_{st}+u_{igst}$$

This regression is run on the same sample of SHGs used for the main results of this paper, those formed on or after 2012. In this regression, τ_gt_ identifies the event year, equaling zero for loans received in the same year as the CIF, 1 for loans received one year after the CIF,-1 for loans received on year before the CIF, and so on. As previously described, *high* is an indicator variable for states with large CIF loan amounts. The regression therefore allows us to examine variation in SHG loans, in years just before and after receipt of CIFs, across states that differ in the amount of CIF funds that they provided to each SHG.

**Fig. 5:**
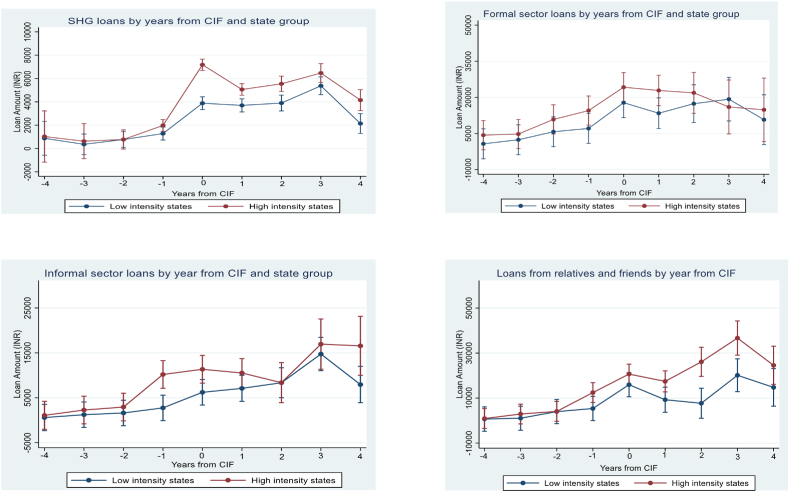
Loan amounts by source, years from CIF and state groups.

The results from the estimation of equation (1) are in Fig. 5. The first panel of this figure provides results for SHG loans. We then replicate this regression for loans from the formal and informal sectors, and for those received from relatives and friends. The data reveal a sharp increase in the amount of SHG loans immediately following the receipt of CIFs, with the loan amounts increasing by approximately INR 2500 in low intensity states, and by approximately INR 5500 in high intensity states. The data also suggest little difference in SHG loan amounts between high and low intensity states prior to the receipt of CIFs. Following this event, loan amounts remain higher in high intensity states, though the difference is less than that in the year in which the CIF was received. This same pattern is not evident for loans from the three alternative loan sources, suggesting that our subsequent estimates of the impact of CIFs are unlikely to reflect any spurious correlation with loans from these other sectors.

### 5.2 Identifying the effect of CIFs on women's decision making

We identify the effect of CIFs on women's bargaining power by exploiting the variation in the receipt of CIFs across SHGs. As in other studies that exploit variation in the phasing of treatment over units, a primary concern is the endogeneity of phasing: SHGs that received CIFs differ from those that do not. The primary reason for this difference is the age of SHGs: older SHGs are more likely to have received CIFs with the average age difference between SHGs with and without CIFs being approximately two years. Second, as previously discussed, variation in the receipt of CIFs reflects variation in state-specific resource constraints as the program scaled. A third possible source of variation is at the SHG level, reflecting the intent of the program to provide CIFs only to SHGs that were functioning well.

Our first approach to the endogeneity of treatment is to condition on observable factors determining access to CIFs and CIF amounts, as in matching methods. We control for variation in state-specific resource constraints through the inclusion of state fixed effects. All regressions also include controls for SHG age. We report results from specifications with a quadratic in SHG age, but also from specifications with indicator variables for the year of formation of the SHG, as well as the interaction of these variables with *high.* This latter interaction allows the effect of SHG age on decision making to vary across state groups, further reducing the possibility that the estimated effect of CIFs is merely capturing variation in SHG age. In these specifications, the variation between SHGs with and without CIFs reflects variation within SHGs of the same age, and hence represents either differences in implementation or differences in SHG attributes. To address the former, we include the previously described controls for the scale of the program across districts and blocks. These scale variables vary across geographies and over time, picking up the effect of geographic and time variation in program implementation.

To eliminate residual differences between SHGs with and without CIFs, we implement a difference-in-difference regression that removes unobservable differences by comparing the difference in outcomes across treatment and control SHGs in states that disbursed large amounts of CIF (*high* intensity states) to this same difference in low intensity states. This regression explicitly controls for selection in the timing of CIFs, allowing for the possibility that those SHGs that received CIFs (conditioning on age) differ from others that did not. It also controls for selection in the amount of CIFs provided by each state.

As is well known, the difference-in-difference regression identifies the effect of CIFs under the assumption that the difference in the decision-making index, prior to the receipt of CIFs by SHGs, across women in SHGs that eventually received CIFs and those that did not in high intensity states equal this same difference in low intensity states. If this assumption is met, then the coefficient on *CIF x high* can be attributed purely to variation in CIF funding across these groups of SHGs. Our basic set of controls for state-level differences, SHG age and scale are required for this assumption. In their absence, the significant differences in program resources and SHG age across high and low intensity states and across SHGs with and without CIFs, and the resulting disparities in SHG quality, are likely to generate corresponding differences in decision-making even in the absence of CIFs.

### 5.3 Basic regression equation and sample

Define *high*_*s*_ = 1 if state s falls in the high CIF group, and let CIF_g_ be an indicator variable for a “treatment” SHG, that is, for SHGs that had received a CIF at the time of our survey. Let W_kg_ be a vector of variables k, k {1,K} that reflect the scale of the program in the district and block at the time of formation of the SHG in question. The basic regression we estimate for the decision making index for a woman in household i, member of SHG g in state s is:

(2) $$dmindx_{igs}=\beta_{0}+\beta_{1}\left(CIF_{gs}xhigh_{s}\right)+\beta_{2}CIF_{gs}+\beta_{3}high_{s}+\underset{s}{\sum }\beta_{4s}shgyrs_{gs}high_{s}+\overset{K}{\underset{k=1}{\sum }}\beta_{5k}\left(W_{kg}*high_{s}\right)+\beta_{6}shgyrs_{gs}+\overset{K}{\underset{k=1}{\sum }}\beta_{7k}W_{kg}+S_{s}+u_{igs}$$

In this regression, S is a set of indicator variables for the 8 states in the survey while *shgyrs* is the number of years since SHG formation. The regression includes interactions of the vector of scale variables, *W,* with *high.* All regressions also include a set of geographical control variables, specifically the population of the block, Gram Panchayat and the village and the distance of the village from the block capital. Standard errors in all regressions are robust to general forms of heteroscedasticity.

The coefficient on the interaction term *CIF x high,* β_1_, identifies the effect of CIFs on the index of women's empowerment under the identifying “common pre-trends” assumption discussed in the previous section.

### 5.4 Testing common trends utilizing the phasing of the program

In programs in which treatment is phased over time and the order of the phased roll-out is known, the difference-in-difference common trends assumption can be tested using the proposed phasing plan across yet-to-be-treated samples. In our context, such a test would require data on three groups of SHGs: those that have already been treated, those who will receive CIFs next, and a third group that will receive treatment last. We mimic this approach utilizing the sample of SHGs who recently received CIFs in the year preceding our survey (2018 and the first few months of 2019).^26^ Under the realistic assumption that behavioral change takes time and that the receipt of just one loan in the first year of the program is unlikely to change a woman's position within the household, this group of recent recipients is equivalent to a control sample that is just about to receive CIFs. The common trends assumption can then be tested by comparing SHGs in this sample (*cif_new)* to those in an older sample of control SHGs without CIFs.

This test is not informative if the estimated coefficient is statistically significant: In this case it is not possible to separate out the effect of pre-trends from a violation of the underlying assumption of this test that the benefits of CIF in the first year were negligible. However, a statistically insignificant coefficient on the interacted term *cif_new x high* will obtain only if both conditions, no pre-trends and no initial effect of CIF, are met.

We support the results of this test with additional regressions using alternative measures of women's decision making determined prior to the formation of SHGs. One such measure is the woman's age at marriage, a variable widely believed to determine her bargaining power within the marriage. Additional support comes from regressions on women's labour force participation rates (LFPRs), an outcome that is also widely viewed as an indicator of women's status, along with their ownership of assets and measures of their involvement in household decisions. For the South Asian economies, the association between women's LFPRs and their socio-economic status, and hence between LFPRs and an index of their decision-making role, is frequently negative (Roy et al., 2015). However, the correlation between these two outcomes, even if negative, suggests that regressions on women's LFPRs will also be informative of the impact of CIFs on women's decision-making. The advantage of LFPRs is that they constitute the one variable for which we have data for the year prior to the onset of NRLP in 2012, enabling a clean test of the equal (pre-program) common trends assumption that underlies the validity of difference-in-difference regressions. These data come from India's 2011 census, which records LFPRs for men and women in every village. We therefore re-run equation (1) using the village female labor force participation rate as the outcome variable, and consider the statistical significance of the interacted term CIF*high. If the identifying assumption is valid, then this term should not be statistically significant.

While the extent to which each of these measures correlates with women's decision-making or empowerment can rightly be questioned, the sum of the evidence from these regressions does help support the robustness of our results.

### 5.5 Falsification tests

We support our interpretation of the regression results through a series of falsification tests. Following Rosenbaum (2005), we report estimates from two types of falsification tests. The first exploits “ineffective treatments”, treatments that should not affect outcomes. In this group, we examine the impact of other SHG treatments or interventions, as well as the impact of alternative groupings of states. For example, states that provided large amounts of CIFs may also be states that differ in terms of other development indicators, calling into question our interpretation of *high* as an indicator of program intensity. To test this, we report regressions that interact *CIF* with indicators derived by grouping states by female literacy rates and the state's proportion of households from scheduled castes and tribes, using data from the 2011 Census. We also assess whether our results are merely picking up the effects of other SHG interventions, focusing on the first infusion of funds into SHGs, the Revolving Funds (RF). Revolving Funds averaged about INR 15,000 per SHG with little variation across states. Because of this lack of variation, an interaction of receipt of RFs with the indicator for high intensity states should have no effect on women's decision making, unless this latter indicator is also picking up the effect of other differences across states that, combined with other SHG interventions, may also affect outcomes.

A second set of falsification tests focuses on “unaffected outcomes,” testing that the coefficient on CIF*high is statistically insignificant for outcomes that should not be affected by the availability of CIFs. A first set of results examines the effect of this interaction term on household variables: the highest level of schooling of adult males and females in the household, the amount of agricultural land owned, and the number of adult males and females. We also report similar regressions that consider the effect of CIF*high on a broad set of SHG attributes (size or total membership, the proportion of members from scheduled castes and tribes, members’ mean years of schooling, the amount of monthly savings).

### 5.6 Supporting evidence from income pooling tests

As noted in Section 2, tests of income pooling commonly used to validate the unitary model provide an alternative method of testing whether CIFs affect household allocations. This section also discussed the assumptions required to interpret any identified effect of CIFs as indicative of their impact on women's bargaining weights. Subject to these concerns, supportive evidence from income pooling tests strengthen our analysis based on the decision-making index. Following the literature, we therefore examine the impact of CIFs on consumption allocations in regressions that condition on total household resources while controlling for their endogeneity.

In addition to their value in supporting our primary results, these regressions allow us to extend the empirical literature to assess whether smaller loans also constitute distribution factors. We do so using data on SHG monthly savings. While research has previously considered multiple distribution factors, this has been in the context of testing whether distribution factors affect allocations only through their effect on women's bargaining weights. Such tests require at least two distribution factors (Browning and Chiappori 2006; Attanasio and Lechene 2014). In contrast, our focus is on whether all enhancements in women's financial access affect bargaining weights. This requires multiple instruments for household savings.

Given our focus on SHG savings we frame our analysis within the context of the empirical literature on lifecycle models that use Frisch or Marginal-utility-of-wealth-held-constant demand functions to incorporate inter-temporal decisions (MaCurdy 1981). MaCurdy (1983) and Blundell and Walker (1986) adapt this framework to cross-sectional data, showing that the marginal utility of wealth can be replaced by the (negative) of household savings, with the endogeneity of savings being recognized. Conditioning on savings, demand functions are only affected by current prices, preference shifters, current preference shocks, distribution factors that affect intra-household bargaining weights and updates to these weights (if participation constraints bind).

Implementing these regressions requires an instrument for savings other than CIFs and SHG monthly savings, one that is uncorrelated with women's bargaining weight. The empirical literature has most commonly used wages or determinants of wages (education, age) as instruments for income (and hence savings), under the assumption that the consumption goods in question are separable from leisure (Attanasio and Lechene 2014). We base identification, instead, on past income shocks, recognizing that current savings reflect past income and past income shocks, and that these variables will affect current outcomes only through savings. More importantly, shocks to income in any given year or season are unlikely to affect a woman's bargaining weight because of their transitory nature and because they affect the totality of household income rather than the woman's resources.

A measure of income shocks is available through survey questions that asked farm households about their expected and realized crop output in the three main agricultural seasons of the previous agricultural year, including for the *Rabi* (winter) season of 2017–18, the year prior to the reference year for our survey.^27^ We use this data to construct an indicator variable for whether output was less than expected in that season.^28^ Combined with SHG monthly savings, the availability of two instruments allows us to implement standard over-identification tests that support their validity.

We first report results from first-stage and reduced form regressions on household savings and consumption allocations respectively, with the set of control variables including *Rabi shock, SHG monthly savings, CIF x high* and the individual components of this interaction. *SHG monthly savings,* the product of the SHG's required monthly saving amount and the number of members of the SHG, represents the funds that would be available to an SHG for internal lending in its first year of operation, prior to the receipt of CIF funds. These regressions also include an expanded set of controls that affect preferences: measures of the demographic profile of the household, captured by the number of household members in 8 gender-age groupings, household size, agricultural land ownership and the highest level of schooling of adult male and female household members.

We then turn to IV regressions that condition on savings, instrumenting it with *Rabi shock*. That is, we re-estimate equation (2), with savings (instrumented) and *SHG monthly savings* included amongst the regressors, using a measure of consumption (described below) as the dependent variable. This regression allows us to test whether *CIF x high* and *SHG monthly savings* affect allocations, even in regressions that condition on household savings. That is, they provide a test of the unitary model and of the hypothesis that only large improvements in a woman's resource position constitute distribution factors that improve a woman's decision-making authority.

In choosing consumption goods for this analysis, a primary concern is that identification assumes that current prices, wages and wage shocks (for this Rabi season) are uncorrelated with individual farmers’ expectations of output and expectational errors from the previous Rabi season.^29^ This condition will be satisfied if prices are determined in larger regional markets and are exogenous for any given farmer. They will, however, be violated if labor markets function imperfectly so that farm households value their time at an endogenously determined shadow or virtual wage that equates the (household) demand and supply for labor. If so, identification requires either the conventional assumption of separability between the sets of goods we consider and leisure or assumptions regarding the correlation between shocks and expectations (conditional on the set of controls) across years.

Our analysis follows the former approach, with attention restricted to purchased goods that are likely to be separable from leisure. This excludes an analysis of food expenditures given the importance of home production in food preparation. Following the literature, we confine our attention to the share of clothing in household expenditures (Browning et al. 1994). The primacy of food in the budget of rural households and the infrequency of expenditures on other items such as furniture, bedding, utensils and minor durables limits the other goods that we can consider; aggregating such goods into a composite will generate results that are difficult to interpret, since the conditions required for aggregation are unlikely to be met.

A second concern, common to other research that implements similar tests of the income pooling model, is that evidence of the statistical significance of *CIF x high* in regressions on household demand, while suggestive of a role for bargaining weights, may also be consistent with other interpretations such as binding labor or credit constraints. However, the availability of an index of women's decision making and evidence of an effect of *CIF x high* on this index suggests that an effect through bargaining weights must exist, even if this does not rule out additional pathways.

## 6 Results

### 6.1 Effect of CIFs on Women's decision making index

Table 2 provides the basic set of results of this paper, reporting coefficients from regressions of the interaction variable *CIF x high* in regressions that vary in the set of controls, including those for SHG year. The first regression is the simplest, including just the interaction variable *CIF x high*, and its individual components (*CIF* and *high*)*,* without any additional controls. The second regression replaces the indicator variable *high* with a set of state fixed effects. The third regression additionally includes SHG years (age) and its interaction with *high.* The next regression represents a far more flexible approach to the incorporation of controls for SHG age, using indicator variables for the year of formation and their interaction with *high.* Regression 5 replaces the indicator variables for SHG age with a quadratic, while the last regression includes the set of controls discussed in the previous section.

**Table 2:** Basic OLS regressions.

	Dependent variable: Women's decision making index					
	(1)	(2)	(3)	(4)	(5)	(6)
CIF*high	−0.02 (0.02)	0.07*** (0.02)	0.07** (0.03)	0.06** (0.03)	0.06** (0.03)	0.07** (0.03)
CIF	0.07*** (0.02)	−0.01 (0.02)	−0.03 (0.02)	−0.03 (0.02)	−0.03 (0.02)	−0.03 (0.02)
High	−0.15*** (0.01)	–	–	–	–	–
SHG years	–	–	0.01** (0.005)	–	−0.03 (0.02)	−0.01 (0.03)
SHG years square	–	–	–	–	0.004* (0.002)	0.002 (0.003)
SHG years * high	–	–	0.003 (0.01)	–	0.10*** (0.04)	0.005 (0.04)
SHG years square * high	–	–	–	–	−0.01*** (0.004)	−0.002 (0.004)
Additional controls	None	state fixed effects	state fixed effects	state FE + SHG year indicators x high	State fixed effects	State fixed effects + additional controls
Sample size	12,548	12,548	12,548	12,548	12,548	12,527
Regression F (Prob > F)	73.74 (0.00)	5.97 (0.00)	5.76 (0.00)	3.56 (0.00)	4.97 (0.00)	4.87 (0.00)

Note: Robust standard errors in parentheses. Additional controls in the last 3 regressions are: district, block and village population, village proportion SC/ST, quadratic in distance to town, distance to block capital, and a set of “scale” variables and their interaction with *high*. These are the number of SHGs in the district (omitting block of SHG location) 2 years prior to SHG formation year, number of villages entered in the district and the block (omitting cluster of SHG location) 2 years prior to SHG formation year.

***Significant at 1% level, **Significant at 5% level, *Significant at 10% level.

The first regression, with no additional controls, corresponds to summary statistics reported in Table 1, revealing a statistically insignificant coefficient on the interaction term *CIF x high*, with large significant effects of its individual components. Specifically, states that awarded large CIF amounts to SHGs are those in which women report lower levels of decision-making responsibility. However, these results differ significantly from those in specifications 2 through 6 that replace the indicator *high* with state fixed effects and include additional controls for SHG age and its interaction with *high.* Though controls for SHG age and their interaction with *high* are statistically significant in several specifications, the results suggest that the primary difference between simple difference-in-difference regression without controls (regression 1) and those reported in columns 2 through 6 stem from the inclusion of state fixed effects. These incorporate the significant differences across states, within the low and high program intensity groups, in the implementation of the program and in the resources devoted to it, differences that are likely correlated with socio-economic characteristics that affect women making even in the absence of CIFs.

The inclusion of state fixed effects also affects the coefficient on *CIF,* which changes from positive and statistically significant in regression 1 (without state fixed effects) to negative and insignificant in regressions with state fixed effects. This change suggests that much of the variation in the timing of CIFs reflects differences in state-level implementation, a difference that varies across the states within each of the two state groupings. While never statistically significant, the negative coefficient on *CIF* in regressions 2 through 6 suggests that the selection of SHGs to receive CIFs early, conditioning on SHG age, targeted relatively disadvantaged SHGs whose members had lower decision-making ability.

The results of this table also reveal no statistically significant variation in the coefficient on *CIF* x *high* across these specifications, attesting to their robustness to variation in the set of control variables. We use the last specification for the remaining results of this paper. This estimate suggests that the provision of CIF in *high* states raises women's decision-making by 0.10, a 2.2% increase over the mean value of 3.08.

To ensure the robustness of our results to alternative ways of constructing the decision making index, Appendix table A reports results using the alternative indices described in sub-section 4.3 while Appendix table B reports results from the sub-indices also detailed in this sub-section. With the exception of decisions regarding what to cook, a decision that may reflect strong cultural norms regarding staple foods, the coefficient on *CIF x high* is statistically significant for each of these sub-indices.

### 6.2 Support for common trends

Table 3 provides support for the common trends assumption underlying the difference-indifference regressions of the previous table, both through a regression analysis on SHGs that vary in their exposure to CIFs as well as through regressions on alternative “baseline” measures of women's empowerment.

**Table 3:** Tests for common trends.

	Dependent variable: DM index		Female labor force participation rate, survey	Village female labor force participation rate, 2011	Women's age at marriage
	(1)	(2)	(3)	(4)	(5)
CIF≤2017 * high	0.16*** (0.04)	0.16*** (0.04)	–	–	–
CIF≥2018 * high group	−0.01 (0.03)	−0.02 (0.03)	–	–	–
CIF * high	–	–	−0.04*** (0.02)	−0.001 (0.01)	0.26 (0.20)
CIF≤2017	−0.09*** (0.02)	−0.09*** (0.02)	–	–	–
CIF≥2018	0.03 (0.03)	0.03 (0.03)	–	–	–
CIF	–	–	0.04*** (0.01)	0.02*** (0.005)	−0.52*** (0.15)
Controls	SHG years, SHG years x high, and additional controls	Quadratic in SHG years, interaction with high + additional controls			
Sample size	12,527	12,527	12,345	12,543	12,409
Regression F (Prob > F)	5.57 (0.00)	5.08 (0.00)	11.76 (0.00)	79.40 (0.00)	11.16 (0.00)

Note: state fixed effects included in all regressions. Robust standard errors in parentheses. Additional controls are: district, block and village population, village proportion SC/ST, quadratic in distance to town, distance to block capital, the set of scale variables and their interaction with *high.*

***Significant at 1% level, **Significant at 5% level, *Significant at 10% level.

The first two columns report results from regressions that divide the sample of SHGs with CIFs into those that received CIFs in the last year (*CIF>=2018)* and those that received CIFs in 2017 or earlier, two years prior to the survey year. Under the assumption that the effect of CIFs on women's bargaining weight takes time to manifest, the coefficient on *CIF>=2018 x high* provides a test for the common trends assumption. The first column reports the results of this test with the set of controls including SHG years and its interaction with *high,* while the second column replaces this with a quadratic in SHG age. Both regressions support the common trends assumption: for SHGs that have only just received CIFs, the coefficient on *CIF x high* is smaller in magnitude and statistically insignificant at conventional levels.

This disaggregation by age significantly increases the estimated impact of CIFs on older SHGs, raising decision making in *high* states by 0.25 (in contrast to the earlier estimate of 0.10), a 8.12% increase.

The remaining three columns test the common trends assumption using alternative correlates of women's decision-making. The first measure is women's labor force participation rate, the one variable for which we have survey data as well as (village level) information from 2011, prior to the onset of the program. Column 3 reveals an effect of CIFs on survey data: the provision of CIFs reduces female labor force participation rates, with the estimated coefficient being statistically significant at the 1% level. In contrast, utilizing village female labor force participation rates from the 2011 census, column (4) suggests no impact of CIFs on baseline values of female labor force participation. Similarly, the last regression, on the age of the woman at the time of marriage, also implies that CIFs had no impact on measures of women's bargaining weights at the time of marriage.

### 6.3 Additional falsification tests

While the results of the previous table support the identification of CIF effects, regressions reported in Table 4 validate the interpretation of estimates as indicative of the effect of CIFs. The first regression replaces the indicator for receipt of CIFs with one for the receipt of the smaller funds provided to SHGs in the form of RFs. The regression reveals that the indicator variable *RF x high* does not significantly affect on women's decision making. This is a strong result, one that suggests that the estimated impact of CIFs is not picking up the effect of other earlier SHG inputs or interventions.

**Table 4:** Falsification tests, Household variables.

	Dependent variable: Women's decision making index			Maximum years of schooling, adult males	Maximum years of schooling, adult females	Agricultural land (acres)	Adult males	Adult females
	Replace CIF with RF, states grouped by CIF amount	States grouped by female literacy rate	States grouped by state proportion SC/ST	CIF, states grouped by CIF amount				
RF * state group	0.04 (0.03)	–	–	–	–	–		
CIF * state group	–	0.01 (0.03)	−0.003 (0.03)	0.16 (0.20)	−0.05 (0.21)	−0.12 (0.08)	0.04 (0.04)	0.03 (0.04)
CIF	–	0.003 (0.02)	0.01 (0.02)	0.27* (0.15)	0.06 (0.15)	0.08 (0.06)	0.03 (0.03)	−0.003 (0.03)
RF	−0.03* (0.02)	–	–	–	–	–	–	–
Sample size	12,527	12,527	12,527	12,543	12,543	12,543	12,543	12,543
Regression F	4.65 (0.00)	4.55 (0.00)	4.45 (0.00	14.23 (0.00)	8.34 (0.00)	10.67 (0.00)	2.42 (0.00)	2.83 (0.00)

Note: state fixed effects included in all regressions. Robust standard errors in parentheses. Additional controls are: quadratic in SHG years and interaction with state group, district, block and village population, village proportion SC/ST, quadratic in distance to town, distance to block capital, the set of scale variables and their interaction with *high.*

***Significant at 1% level, **Significant at 5% level, *Significant at 10% level.

The next two regressions address a primary concern with our methodology, that state groupings may just be reflecting broad state-level differences in economic conditions and are not reflective of variations in the magnitude of CIF funding and hence of the intensity of the CIF input. These regressions reveal that replacing the indicator *high* with alternative indicators based on the state's female literacy rate or the state's proportion of the population from scheduled castes and tribes does not have a similar effect. As with the regression on RFs, this provides strong support that *CIF x high* does, in fact, capture variation in the intensity and incidence of the program.

The remaining 5 columns test the effect of this interaction on “unaffected outcomes” described in the previous section, those that should not be affected by the provision of CIFs. Conversely, if the interaction variable *CIF x high* merely reflects geographic variation in socio-economic conditions, one would expect it to effect at least some of these household attributes. The results of the estimation of our basic regression, with controls, on this broad range of outcomes provides further support for our results: The provision of CIFs does not determine any of these outcomes.

Finally, regressions reported in Table 5 repeats this last set of tests on SHG attributes, using data on the 2568 survey SHGs from the SHG module. Though these attributes were measured at the time of the endline survey, they reflect variables that are determined at the time of SHG formation and hence are likely to change only if membership significantly changes over time. The results reveal no effect of *CIF x high* on SHG attributes.

**Table 5:** Falsification tests, SHG attributes.

	SHG size	Prop SC/ST members	Member's mean years of schooling	Amt of monthly savings (INR)
CIF x high	0.15 (0.13)	−0.04 (0.03)	0.21 (0.18)	−1.16 (4.80)
CIF	0.08 (0.09)	−0.03 (0.02)	0.04 (0.11)	−1.07 (4.25)
Sample size	2568	2568	2568	2568
Regression F	3.16 (0.00)	85.76 (0.00)	6.25 (0.00)	3.42 (0.00)

Note: State fixed effects included in all regressions. Robust standard errors in parentheses. Additional controls are: quadratic in SHG years and interaction with *high,* district, block and village population, village proportion SC/ST, quadratic in distance to town, distance to block capital, scale variables and their interaction with *high.*

***Significant at 1% level, **Significant at 5% level, *Significant at 10% level.

Taken together, the results of our regression test for common trends as well as this extensive set of falsification tests suggest strong support for our empirical methodology and for our interpretation of the coefficient on *CIF x high* as indicative of the effect of CIFs on women's intra-household bargaining weight.

### 6.4 Results from instrumental variables savings-conditioned regressions

In this section, we report results from regressions that implement the instrumental variable methodology described in the previous section to test the effect of household savings and the relative importance of small versus large increments in women's financial resources on decision-making and household demands. All regressions include the set of controls used in the previous regressions as well as the preference shifters described in Section 5.

Column 1 reports results from first stage regressions on household savings, revealing a strong statistically significant effect of *CIF x high* and *Rabi shock,* as well as that of SHG monthly contributions (significant at the 5% level). Savings fall with higher SHG monthly savings, consistent with the hypothesis that increases in a SHGs internal savings enable small loans that facilitate increases in household expenditures. Savings also fall with negative income shocks. In contrast, *CIF x high* has a strong statistically significant effect on household savings, suggestive of a positive effect of large loans on household income through the investments in productive assets that they enable.

Results from instrumental variable regressions on clothing shares are in columns three through five.^30^ Column three reports results from regressions that instrument savings with *Rabi shock,* with SHGs' monthly savings and *CIF x high* included as regressors. This regression reveals that SHGs' monthly savings have an insignificant effect on clothing shares in regressions that condition on savings, suggesting that any impact on household allocations comes only through its impact on total household resources. In contrast, the statistically significant coefficient on *CIF x high* in this same regression suggests that CIFs affect household allocations independently of their effect on savings confirming that large improvements in women's financial access have effects on households that differ from those that would result from a general improvement of the household to financial resources. These differences suggest that who controls household resources only matters if the amounts in question are relatively large.

The insignificant effect of *SHG monthly savings* on clothing shares in this regression combined with its positive effect on total household savings (Table 6) allows us to validate our identification of household savings, using a standard over-identification test. We therefore instrument household savings by *SHG savings amount* and include *Rabi shock* amongst the regressors. The results, reported in the panel titled *Regression 2*, confirm the validity of *Rabi shock* as an instrument for savings. This specification yields coefficients on household savings and *CIF x high* that are statistically equivalent to those reported in the top panel, though a loss of precision reduces the statistical significance of *CIF x high.*

**Table 6:** First stage, reduced form and IV regressions.

Variable	Household savings (INR′000)	Clothing Share (Reduced form)	IV	IV	IV
	(1)	(2)	(3)	(4)	(5)
Household savings (INR ‘000)	–	–	0.02*** (0.004)	0.03** (0.01)	0.02*** (0.003)
CIF x high	12.44*** (4.34)	0.62*** (0.20)	0.42** (0.20)	0.31 (0.25)	0.40** (0.20)
Rabi shock	−25.32*** (2.35)	−0.42*** (0.10)	–	0.21 (0.31)	–
SHG monthly savings	−0.63** (0.28)	−0.02*** (0.005)	−0.01 (0.01)	–	–
CIF	−7.93** (3.21)	−0.31** (0.14)	−0.18 (0.14)	−0.11 (0.17)	−0.17 (0.14)
Regression F/Chi square	28.62 (0.00)	16.8 (0.00)	9.89 (0.00)	8.76 (0.00)	10.10 (0.00)
Sample size	11,967	11,967	11,967	11,967	11,967

Note: Robust standard errors in parentheses. Additional regressors are: highest adult male and female education years; agricultural land ownership; 8 gender-age demographic categories and household size; indicator for scheduled caste and tribes; interactions of *high* with a quadratic in SHG age and with a set of scale variables; district, block, and village population; village proportion SC/ST; quadratic in distance of village from nearest town, distance from block capital, and state dummy variables.

***Significant at 1% level, **Significant at 5% level, *Significant at 10% level.

Finally, column 5 uses both *SHG monthly savings* and *Rabi shock* as instruments for household savings, reporting coefficients on total household savings and *CIF x high.* As in other specifications, the results reveal that household allocations reflect both total household savings and women's access to large financial resources. Our results thus suggest that women's access to large funds constitutes a distribution factor, shifting expenditure patterns even in regressions that condition on the household's total resources.

## 7 Conclusion

This paper provides empirical support for the hypothesis that improvements in women's decision-making ability over the course of a marriage require large improvements in their access to financial resources. This is the prediction of a theoretical literature which postulates that women who enter a marriage with very low bargaining power are unlikely to see any improvement in their status as a consequence of policies that have only marginal effects on their reservation utility. For such women, change requires large improvements in their resource base.

Our research is based on an analysis of Self Help Groups supported by India's National Rural Livelihoods Mission. The program supported the development of small self help groups comprising women members. As is typically the case, SHGs provided a means for women to aggregate monthly savings into amounts that formed the basis for small internal loans. However, the program also enabled significantly larger loans through the provision of Community Investment Funds, CIFs. Utilizing variation in the phased delivery of CIFs and in CIF amounts across states, we identify the effect of CIFs using a difference-in-difference methodology. We subject our results to several specification and robustness tests. All regressions document a significant effect of CIFs on women's decision-making, suggesting that one explanation for the mixed evidence in the existing literature regarding the impact of SHGs and microfinance institutions on measures of women's decision-making relates to the magnitude of funds available to these groups.

Cognizant of the difficulties in interpreting results based on indices of women's decision-making, we also report results from regressions that examine the explanatory power of CIFs in regressions that condition on the household's total resources (savings). We confirm that access to these large funds serve as a distribution factor, affecting household allocations directly. Conversely, though access to small loans affects household savings, the effect on household allocations occurs only through household savings. Put differently, financial inclusion policies that target women produce results that differ from those that target households only if they provide women with access to relatively large loans.

Our results have significant implications for policy, highlighting the need to pay attention to quantities, rather than to implement financial inclusion policies without regard to the loan amounts that are available through them.

## Author statement

**Kochar:** Conceptualization; Formal analysis; Methodology; Writing of original draft and revisions. **Nagabhushana:** Conceptualization; Data curation; Investigation. **Sarkar:** Conceptualization; Investigation; Project administration. **Shah:** Conceptualization; Data curation; Investigation; Validation. **Singh:** Conceptualization; Methodology; Validation; Writing of revisions.

## Figures and Tables

**Table:** 

	High control	PCA	Mean index, omit land, weddings	Mean index from (3), also omit moneylender and bank loans
	(1)	(2)	(3)	(4)
CIF*high	3.33*** (1.25)	0.64*** (0.21)	0.07** (0.03)	0.07** (0.03)
CIF	−3.21*** (0.99)	−0.20 (0.17)	−0.03 (0.02)	−0.03 (0.02)
Sample size	12,527	8471	12,527	12,527
Regression F (Prob > F)	6.29 (0.00)	3.85 (0.00)	4.88 (0.00)	4.93 (0.00)

Note: state fixed effects included in all regressions. Robust standard errors in parentheses. The high control index assigns a value 1 if the woman reports having major control or sole control over the decision in question, and reports the mean of this indicator variable across all categories. PCA uses principal components to calculate the index. The third column uses the mean index used for the regressions of this paper, but omitting decisions reporting to weddings and the sale of land, while the last column additional removes decisions regarding bank and moneylender loans. Additional regressors: interactions of *high* with a quadratic in SHG age and with a set of scale variables; district, block, and village population; village proportion SC/ST; quadratic in distance of village from nearest town, distance from block capital.

***Significant at 1% level, **Significant at 5% level, *Significant at 10% level.

**Table:** 

Decision	Coefficient on		Regression F	Sample size
	CIF x high	CIF		
Food expenditure	0.11*** (0.04)	−0.08*** (0.03)	10.62 (0.00)	12,476
Food – what to cook	0.02 (0.04)	−0.03 (0.03)	9.26 (0.00)	12,496
Children's expenses	0.08** (0.03)	−0.05** (0.02)	3.83 (0.00)	12,198
Clothing	0.07** (0.03)	−0.04* (0.02)	4.32 (0.00)	12,511
Durables	0.13*** (0.03)	−0.04* (0.02)	7.05 (0.00)	12,368
Loans	0.10** (0.04)	−0.08*** (0.03)	5.99 (0.00)	12,534

Note: Robust standard errors in parentheses. Additional regressors are listed in Appendix table A.

***Significant at 1% level, **Significant at 5% level, *Significant at 10% level.

## Data Availability

Data will be made available on request.

## Footnotes

1 Brody et al. (2017) note that low average returns mask heterogeneity across SHGs that differ in terms of their design, the socio-economic characteristics of their numbers and the geographies they cover. Available reviews do not assess variation in returns by loan size.

2 The large literature on this topic is reviewed in Azariadis (1996), Banerjee (2003) and Piketty 2000).

3 The BRAC program (Bandiera et al., 2017) provided ultra poor women with assets that doubled their baseline wealth, with the total cost of the benefits provided to each woman being approximately US $1120 (in 2007 PPP terms).

4 In 2019, the World Bank augmented the program with additional resources, creating the National Rural Economic Transformation Project (NRETP).

5 Based on the 2020 wage in India's national workfare program, the Mahatma Gandhi National Rural Employment Guarantee.

6 Duflo (2001) evaluates the effect of a school construction program by comparing outcomes across cohorts in regions with varying program intensity. Jacoby (2002) exploits cross-sectional variation in program intensity to assess school meals, comparing outcomes on school and non-school days.

7 Our thanks to the editor for bringing this point to our attention.

8 Lise and Yamada (2019) provide empirical support.

9 Details of the program available in Government of India (2017). The program was targeted at all households that suffered from at least one measure of “deprivation” as identified by India's 2011 Socio-economic Caste Census.

10 These amounts are based on data for all loans provided by SHGs and the total number of loans since SHG formation, collected in the SHG module.

11 Though CIFs were to be provided to SHGs through VOs, the program allowed funds to be directly provided to SHGs who had not yet been federated into VOs.

12 The percentage of central to state funds was set at 75:25 for all major states at the start of the program, but was revised to 60:40 in 2015–16.

13 Rajasthan's Annual Action Plan 2014–15 explains the slow growth in the number of “Internal Resource Persons” responsible for forming SHGs, VOs and CLFs as follows: “The capacity building of internal CRP teams is process intensive and it takes lot of time and resources. Deployment of internal CRPs got delayed because of this. In turn capacity building activities of community institutions could not be carried out as planned.” Annual Action Plans for 2015–16 for the Jharkhand and Uttar Pradesh similarly identified shortages of community cadre members as binding constraints.

14 These states are: Bihar, Chattisgarh, Jharkhand, Madhya Pradesh, Maharashtra, Rajasthan, Odisha and Uttar Pradesh.

15 Details of the survey design and methodology are in Kochar et al., (2020).

16 In the state of Madhya Pradesh, the normal pattern of phasing across blocks, clusters and villages was not observed for NRLP blocks, but was implemented for a set of blocks implementing an earlier World Bank program (DPEP) that followed the same guidelines. This meant that the Madhya Pradesh sample included SHGs that were formed between 2009 and 2012 with different guidelines for the receipt of CIFs. Additionally, the older age of SHGs from this state also meant that it was characterized by a relatively high percentage of “defunct” SHGs, those that existed in MIS records but were not functioning at the time of the survey (as was determined by field level teams).

17 Specifically, our study is restricted to households with at least one male member over the age of 15.

18 The alternative of measuring savings using data on transactions in assets was not possible, since this information is not available for all assets.

19 We use the state-specified norms for CIFs rather than the amounts reported by SHGs to minimize endogeneity concerns.

20 Of 17 quantitative studies that analyzed the impact of SHGs on decision-making reviewed in Brody et al. (2017), 11 used similarly constructed indices.

21 These were expenditures on: food; kitchen items; fuel; household durables; clothing for men, women and children; and weddings and other ceremonies; choice of schools for children; health care providers (for women, men and children); home improvements including construction of toilets; sale of land; stocks of foodgrains; and the acquisition of loans from different sources (SHGs, formal institutions, information institutions and relatives and friends).

22 In turn a function of its large scale which precluded a more qualitative approach.

23 This amount was INR 110,000 in early years of the program but fell of slightly in 2018 and 2019.

24 Cattaneo et al. (2016) show that estimates from this regression represent a weighted average across cut-offs of local average treatment effects.

25 Observations of loans 3 years after receipt of CIFs come from CIFs received in 2016 or earlier while observations for loans received 3 years before CIFs come from those received in 2015 or later.

26 This requires data on the year of receipt of CIFs. This information was collected in our survey.

27 Because we measure savings as the difference between income and expenditure, this also means that our measure of savings uses income data from the Rabi season of 2017–18, rather than that from 2018–19. Thus, while savings always reflects past income, in our case the measure of savings is explicitly based on income from the previous Rabi season. On the assumption that seasonal income follows a random walk, income in the current Rabi season equals income in the previous season and an expectational error, which is then a component of the regression residual.

28 The use of determinants of agricultural incomes for instrument implies that we identify agricultural incomes and not the incomes of non-agricultural households. The strength of this instrument derives from the fact that 66% of farmers report ownership of agricultural land.

29 Actual output, including any expectation error, will affect current Rabi output. This is included in savings and hence is not a concern. The concern, instead, is that previous shocks affect other determinants of savings-conditioned demand equations, that is prices, wages and preference shocks.

30 Clothing share are expressed as percentages of expenditure rather than as shares so as enhance exposition in the tables.
